# Machine learning-based early prediction of multiple chronic disease risk in aging Chinese population: A longitudinal analysis using CHARLS data

**DOI:** 10.1097/MD.0000000000049175

**Published:** 2026-06-05

**Authors:** Yu Wang

**Affiliations:** a Zhengzhou Health College, Zhengzhou, Henan, China.

**Keywords:** CHARLS, health management, machine learning, public health, risk prediction

## Abstract

Population aging has intensified the burden of multimorbidity among older adults, necessitating effective tools for the early identification of high-risk individuals. This retrospective cohort study utilized data from 8552 participants in the China Health and Retirement Longitudinal Study (2011–2018) to develop and validate machine learning models for predicting incident multimorbidity, defined as the new-onset co-occurrence of 2 or more chronic conditions among participants free of multimorbidity at baseline, assessed across 14 physician-diagnosed diseases. Five algorithms, including logistic regression, random forest, extreme gradient boosting, support vector machine, and k-nearest neighbors, were compared, with temporal validation conducted using an independent cohort of 3218 participants from the 2013 China Health and Retirement Longitudinal Study wave. Extreme gradient boosting achieved the best discrimination (area under the receiver operating characteristic curve = 0.803 in testing, 0.779 in temporal validation) with acceptable calibration (Hosmer-Lemeshow *P* = .189). Baseline chronic condition status, age, self-rated health, and depressive symptoms were the most influential predictors, with health status indicators collectively contributing the largest proportion of predictive importance. Machine learning algorithms can effectively stratify multimorbidity risk in aging Chinese populations, and the identified predictive factors offer potential directions for risk-focused surveillance and preventive strategies in primary care settings.

## 1. Introduction

Aging populations have become an important demographic change with profound effects on public health systems worldwide. Systematic reviews on the prevalence of multimorbidity among Chinese populations have noted an alarmingly high prevalence among older populations, reaching proportions requiring urgent attention from policymakers.^[1]^ Furthermore, the Global Burden of Disease Study 2019 confirms that noncommunicable diseases account for an increasing proportion of disability-adjusted life years globally, with comorbid conditions further aggravating health outcomes.^[2]^ The Peking University–Lancet Commission message emphasizes that China finds itself at a most critical juncture because it has an older generation and a healthcare system that needs intense changes.^[3]^ Studies have shown remarkable regional variability in disease prevalence among different socioeconomic and geographically disparate populations, and longitudinal research based on national samples has identified significant factors influencing comorbidity rates based on educational status, income, smoker status, exercise status, and health access.^[4–7]^ Comorbidity has become increasingly recognized as a risk factor associated with accelerated functional decline, elevated mortality, and higher healthcare utilization, underscoring the need for early identification and prioritization of high-risk patients.^[8,9]^

Machine learning (ML) approaches have shown considerable promise in creating valid predictive models on chronic disease outcomes, often outperforming conventional statistical models on discrimination and calibration, especially when working with large and complex datasets.^[10]^ The capacity of these models to detect and exploit interactions and nonlinear correlations among variables makes them highly appropriate for capturing the multicausal etiology associated with multimorbidity.^[11]^ Recent developments have shown that ensemble learning approaches will be useful for specific forms of prediction within chronic disease, but concerns regarding interpretability and clinical relevance have been raised.^[12,13]^ Despite these advances, issues with measurement heterogeneity among different concepts of multimorbidity have been suggested as influencing cross-study comparability and the establishment of more unified prediction tools among studies.^[14,15]^ The aims of the current research endeavor lie in developing and testing prediction models based on ML for incident multimorbidity among mid-to-late life members of Chinese populations using longitudinal data sets available from the China Health and Retirement Longitudinal Study (CHARLS). By employing and testing 5 algorithm-based approaches, including logistic regression, random forest, extreme gradient boosting (XGBoost), support vector machines (SVMs), and k-nearest neighbors, it will be determined how best these methods might be adapted for prediction, and insights will be provided on certain factors influencing multimorbidity onset.

## 2. Methods

### 2.1. Study design and data source

The current retrospective cohort analysis uses data from the CHARLS, a nationwide survey with a longitudinal sample of Chinese adults 45 years old and older that obtained information on health and economic and social conditions.^[5]^ The research method aligns with the Strengthening the Reporting of Observational Studies in Epidemiology guidelines.^[16]^ CHARLS adopted a multistage stratified probability proportionate-to-size sample, involving 150 counties and covering 28 provinces in mainland China. The sample followed a cohort enrolled in 2011 with bi-annual follow-ups conducted up to 2018, enabling estimation of the buildup of multimorbidity within 7 years.^[5,17]^ The inclusion criteria were participants 45 years and older at baseline, having full data on chronic disease without developing multimorbidity at enrollment; and the exemption criteria included excessive missing data, loss to follow-up, and cognitive impairment. Of 17,708 participants at enrollment, 8552 qualified as subjects for the main analysis cohort, and 3218 subjects from the 2013 cohort enrollment qualified for an independent temporal validation cohort.

### 2.2. Variable definition

The main measure was incident multimorbidity, operationalized as the cumulative incidence of 2 or more comorbidities among participants who initially had fewer than 2 comorbidities at baseline.^[18]^ The cutoff represents the most commonly adopted benchmark within the research area, ensuring consistency with previous research despite recognized variability across different studies.^[19]^ Outcomes were ascertained for 14 comorbidities, all of which were documented consistently across CHARLS waves and included hypertension, diabetes, cancer, chronic lung disease, heart disease, stroke, kidney disease, liver disease, digestive disease, psychiatric disorders, memory disease due to neurological problems, arthritis, asthma, and dyslipidemia, ascertained via physician-defined diagnoses as reported by participants.^[20]^ Potential predictors were classified into 5 categories: demographic/socioeconomic factors (age, sex, marital status, education, and residence), socioeconomic factors (household income quintile, type and presence of insurance, and employment status), lifestyle variables (current smoker status, alcohol consumption, and physical activity), health status variables (body mass index [BMI], subjective health status, disability due to physical limitations, and depression), and initial burden of disease at study entry (number of comorbidities at entry).

### 2.3. ML model development

**Figure 1.**
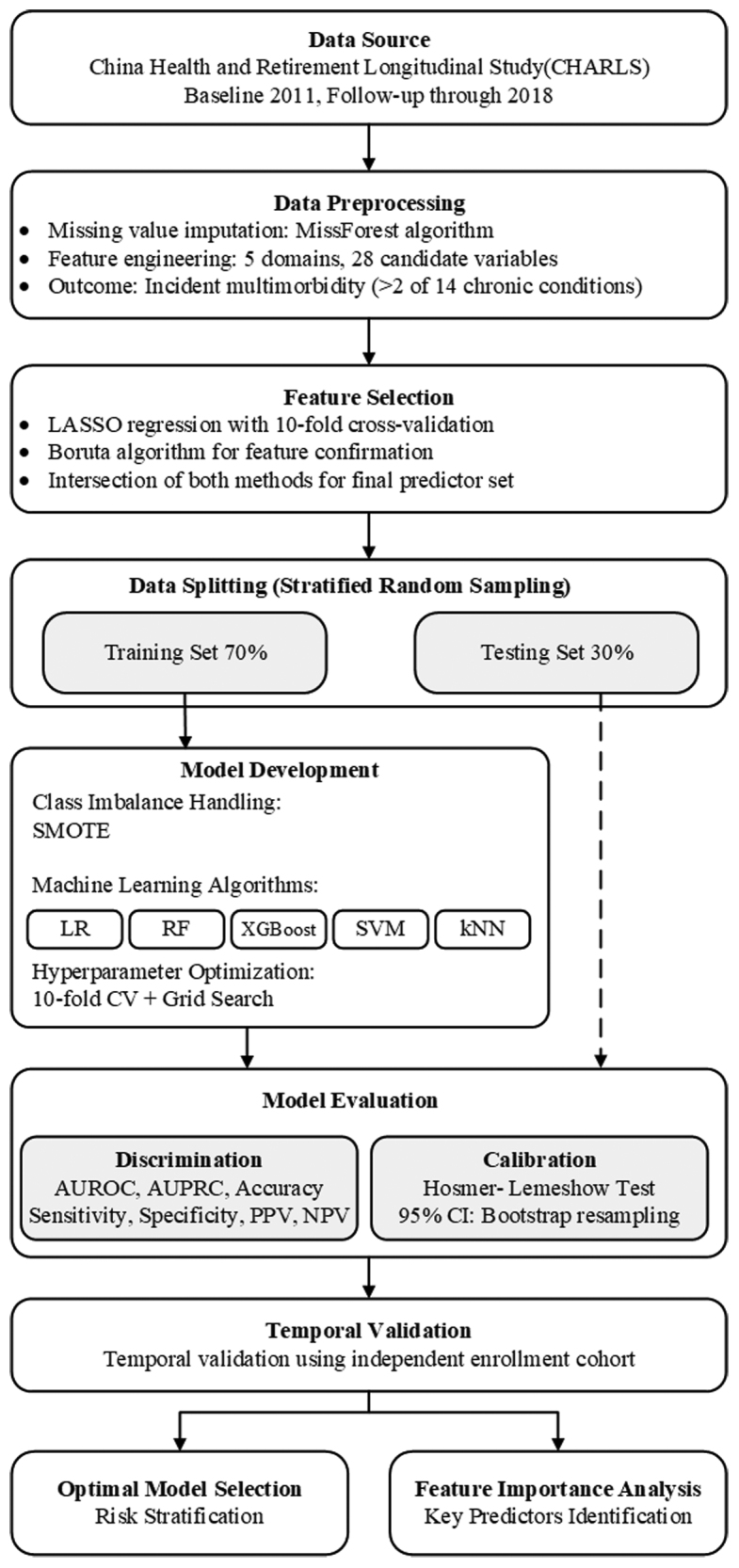
Flowchart of the machine learning modeling framework for multimorbidity prediction. AUPRC = area under the precision-recall curve, AUROC = area under the receiver operating characteristic curve, CI = confidence interval, kNN = k-nearest neighbor, NPV = negative predictive value, PPV = positive predictive value, SMOTE = synthetic minority oversampling technique, SVM = support vector machine, XGBoost = extreme gradient boosting.


The analytical pipeline, as shown in Figure 1, included structured preprocessing that addressed data quality issues. Handling missing data involved the MissForest algorithm, an iterative and nonparametric technique using predictions from random forest models. The extent of missingness varied across predictor variables, with the proportion of missing values ranging from 0% to 8.3% across all 28 candidate variables. Variables with the highest missingness included household income quintile (8.3%), BMI (6.2%), and physical activity (4.6%), while sociodemographic variables including age, sex, and residence exhibited missing rates below 1.0%. The missing data mechanism was assumed to be missing at random, consistent with the characteristics of population-based longitudinal survey data of this nature.^[21]^ The least absolute shrinkage and selection operator (LASSO) algorithm based on L1-regularization^[22]^ was employed for feature selection. Because the variable for multimorbidity had a binary response, LASSO regularized a logistic regression model with a penalty term incorporated into a log-likelihood equation as follows:

$$\hat{\beta }=\mathrm{\backslash arg}\max_{\beta }\left\{\sum_{i=1}^{n}\left[y_{i}\log(\pi_{i})+(1-y_{i})\log(1-\pi_{i})\right]-\lambda \sum_{j=1}^{\;p}|\beta_{j}|\right\}$$ (1)

where $\pi_{i}=\frac{1}{1+\exp(-\overset{T}{\underset{i}{x}}\beta )}$ represents the predicted probability of multimorbidity for individual *i*, *y*_*i*_ denotes the observed binary outcome, *x*_*i*_ is the vector of predictor variables, β represents the coefficient vector, λ is the regularization parameter controlling the strength of the L1 penalty, and *p* indicates the total number of predictors. The optimal value of λ was determined through 10-fold cross-validation maximizing the area under the receiver operating characteristic curve (AUROC). Complementary feature selection using the Boruta algorithm, which assesses variable importance against randomly permuted shadow features, provided confirmatory evidence for the robustness of selected predictors.^[23,24]^ The intersection of variables identified by both methods formed the final feature set for model training.

The problem of class imbalance was remedied by using synthetic minority oversampling technique (SMOTE) solely on the training set.^[25]^ The sample was split into a training set and a testing set using stratified random sampling, with 70% assigned to the training set and 30% assigned to the testing set. Five methods were compared: a linear method based on logistic regression, a bagging method based on random forest classification,^[26]^ an extreme gradient boosting method based on the XGBoost regularized sequential learning algorithm,^[27]^ SVMs with a radial basis function kernel, and k-nearest neighbors methods. Hyperparameter optimization employed exhaustive grid search with 10-fold cross-validated AUROC as the selection criterion. The search space and final optimized values for each model were as follows: for logistic regression, the regularization parameter C was searched over {0.001, 0.01, 0.1, 1, 10, 100}, with a final optimized value of 1.0. For random forest, the number of estimators was searched over {100, 200, 300}, maximum tree depth over {5, 7, 10, None}, and minimum samples per leaf over {1, 2, 5}, yielding final values of 200, 10, and 2, respectively. For XGBoost, the search space encompassed learning rate {0.01, 0.05, 0.1, 0.2}, maximum depth {3, 5, 7}, number of estimators {100, 200, 300}, subsample ratio {0.7, 0.8, 1.0}, and column subsampling ratio {0.7, 0.8, 1.0}, with final optimized values of 0.05, 5, 200, 0.8, and 0.8, respectively. For SVM, the regularization parameter C was searched over {0.1, 1, 10, 100} and the kernel coefficient gamma over {scale, auto, 0.001, 0.01, 0.1}, with final values of 10 and scale, respectively. For k-nearest neighbors, the number of neighbors k was searched over {3, 5, 7, 9, 11, 15, 21}, yielding a final value of 9.

### 2.4. Model evaluation

Model performance was assessed using various metrics. AUROC, area under the precision-recall curve, accuracy, sensitivity, specificity, positive predictive value, and negative predictive value were some metrics used for evaluating the performance of various algorithms on DIS criteria.^[28]^ AUROC values above 0.7 were recognized as representing acceptable discriminative performance, and values exceeding 0.8 were recognized as representing good discriminative performance.^[29]^ Calibration was assessed using the Hosmer-Lemeshow goodness of fit test and calibration curves plotting observed event rates against mean predicted probabilities across risk deciles for both the internal testing set and the temporal validation cohort. Decision curve analysis was performed to evaluate the net clinical benefit of each model across a range of probability thresholds, comparing model-based decision strategies against the treat-all and treat-none reference strategies. To enhance model interpretability, SHapley Additive exPlanations (SHAP) values were computed for the optimal XGBoost model using a TreeExplainer, quantifying the marginal contribution of each predictor to individual-level predictions and providing a theoretically grounded complement to gain-based feature importance metrics.^[30]^ Statistical uncertainty is quantified using 95% confidence intervals (CIs) obtained from bootstrapping resampling with 1000 resamples. Feature importance was quantified using algorithm-specific metrics, including regression coefficients for logistic regression, mean decrease in Gini impurity for random forest, and gain-based importance for XGBoost. SHAP values were additionally computed for the XGBoost model to decompose predicted probabilities into individual feature contributions, enabling both global feature ranking and local explanation of individual predictions. As a sensitivity analysis, a complete-case analysis was conducted by reestimating the XGBoost model on participants with no missing values on any of the 15 selected predictors, without applying MissForest imputation, to assess the robustness of the primary findings to the missing data handling approach. All analyses were conducted in Python 3.9.7. Key libraries included scikit-learn (v1.2.2) for model development and evaluation, XGBoost (v1.7.5) for gradient boosting implementation, imbalanced-learn (v0.10.1) for SMOTE oversampling, missingpy (v0.2.0) for MissForest imputation, and numpy (v1.23.5) and pandas (v1.5.3) for data processing. The complete analytical code is publicly available at https://github.com/mamingqian78-droid/multimorbidity-prediction-CHARLS.

## 3. Results

### 3.1. Study population and baseline characteristics

Applying predefined inclusion and exclusion criteria to the CHARLS 2011 Baseline Survey resulted in a sample size of 8552 respondents who were not presenting with multimorbidity at baseline and had complete longitudinal follow-up data up to 2018. The criteria for exclusion among 17,708 individuals who participated at baseline included prevalent multimorbidity (n = 4521, 25.5%), missing data on chronic illnesses (n = 2847, 16.1%), lost-to-follow-up subjects (n = 1432, 8.1%), and excessive missing demographic information (n = 356, 2.0%). The data set was stratified and randomly split into a training set (n = 5986, 70%) and a testing set (n = 2566, 30%). A separate temporal validation set (n = 3218) was constructed from the 2013 CHARLS enrollment cohort.

During a period of 7 years, 2463 participants experienced the onset of multimorbidity, and the cumulative incidence rate was 28.8% (95% CI = 27.8%–29.8%). Missing data on predictor variables were imputed using the MissForest algorithm. The proportion of missing values per variable ranged from 0% to 8.3%, with household income quintile (8.3%), BMI (6.2%), physical activity (4.6%), depressive symptoms (3.5%), and alcohol consumption (2.8%) exhibiting the highest rates of missingness; detailed variable-level missing data proportions are presented in Table S1, Supplemental Digital Content. Imputation accuracy was satisfactory, as indicated by an out-of-bag error rate of 0.127 for continuous variables and 0.089 for categorical variables, both of which fall within acceptable bounds for this class of imputation method. Baseline variables were summarized stratified by outcome status in Table 1. Those who experienced multimorbidity were significantly older, with a median age of 61.3 versus 55.8 years (*P* < .001), with higher proportions of females (57.0% vs 51.0%, *P* < .001), and with greater proportions of obesity (16.9% vs 10.0%, *P* < .001), poor self-reported health status (60.8% vs 40.0%, *P* < .001), and depression (42.9% vs 29.0%, *P* < .001). The presence of chronic conditions at baseline significantly differentiated these 2 groups (59.8% vs 32.0%, *P* < .001).

**Table 1:** Baseline characteristics of study participants by multimorbidity status.

Characteristic	Total (n = 8552)	Non-multimorbidity (n = 6089)	Multimorbidity (n = 2463)	*P* value
Sociodemographic factors				
Age, years, mean (SD)	57.4 (9.2)	55.8 (8.7)	61.3 (9.4)	<.001
Female, n (%)	4512 (52.8)	3108 (51.0)	1404 (57.0)	<.001
Married/partnered, n (%)	7268 (85.0)	5243 (86.1)	2025 (82.2)	<.001
Rural residence, n (%)	5302 (62.0)	3774 (62.0)	1528 (62.0)	.952
Education level, n (%)				
No formal education	2736 (32.0)	1779 (29.2)	957 (38.9)	<.001
Primary school	2651 (31.0)	1888 (31.0)	763 (31.0)	
Secondary school	2394 (28.0)	1827 (30.0)	567 (23.0)	
Higher education	771 (9.0)	595 (9.8)	176 (7.1)	
Socioeconomic factors				
Lowest income quintile, n (%)	1710 (20.0)	1096 (18.0)	614 (24.9)	<.001
Health insurance coverage, n (%)	7953 (93.0)	5663 (93.0)	2290 (93.0)	.876
Currently employed, n (%)	5987 (70.0)	4446 (73.0)	1541 (62.6)	<.001
Lifestyle factors				
Current smoker, n (%)	2479 (29.0)	1827 (30.0)	652 (26.5)	.001
Regular alcohol use, n (%)	1881 (22.0)	1400 (23.0)	481 (19.5)	<.001
Physical inactivity, n (%)	3078 (36.0)	2009 (33.0)	1069 (43.4)	<.001
Health status indicators				
BMI, kg/m^2^, mean (SD)	23.6 (3.8)	23.2 (3.6)	24.5 (4.1)	<.001
BMI ≥ 28 kg/m^2^, n (%)	1026 (12.0)	609 (10.0)	417 (16.9)	<.001
Poor/fair self-rated health, n (%)	3933 (46.0)	2436 (40.0)	1497 (60.8)	<.001
ADL limitation, n (%)	513 (6.0)	243 (4.0)	270 (11.0)	<.001
Depressive symptoms (CESD-10 ≥ 10), n (%)	2822 (33.0)	1766 (29.0)	1056 (42.9)	<.001
Baseline chronic disease				
One chronic condition at baseline, n (%)	3421 (40.0)	1949 (32.0)	1472 (59.8)	<.001

ADL = activities of daily living, BMI = body mass index, CESD-10 = 10-item Center for Epidemiological Studies Depression Scale, SD = standard deviation.

*P* values were derived from chi-square tests for categorical variables and independent *t* tests for continuous variables.

### 3.2. Model performance comparison

The feature selection task was carried out using LASSO regression with 10-fold cross-validation, and an optimal value for the penalty term λ of 0.023, with 18 attributes selected. Boruta made a similar identification, with 16 features being more significant compared to shadow attributes. The common set among these methods included 15 stable variables useful for model development. To correct imbalances, the SMOTE technique was employed on the training set.

Five models were trained and validated on both the internal testing set and the temporal validation cohort. A comparison of performances is shown in Table 2. XGBoost showed better discriminatory capacity, with an AUROC value of 0.803 (95% CI = 0.784–0.822) on internal testing data, outperforming random forest (0.786, 95% CI = 0.766–0.806), SVM (0.761, 95% CI = 0.740–0.782), logistic regression (0.738, 95% CI = 0.716–0.760), and k-nearest neighbors (0.714, 95% CI = 0.691–0.737). The ranks remained consistent in the temporal validation cohort, with AUROC value of 0.779 (95% CI = 0.758–0.800), representing a 3.0% relative reduction and acceptable model transportability. Precision-recall curves validated better performance of XGBoost with area under the precision-recall curve = 0.612.

**Table 2:** Performance comparison of machine learning models in predicting multimorbidity.

Model	Dataset	AUROC (95% CI)	AUPRC (95% CI)	Accuracy	Sensitivity	Specificity	PPV	NPV
Logistic regression	Testing	0.738 (0.716–0.760)	0.524 (0.489–0.559)	0.682	0.654	0.693	0.463	0.832
	Temporal validation	0.721 (0.698–0.744)	0.507 (0.471–0.543)	0.668	0.638	0.680	0.451	0.824
Random forest	Testing	0.786 (0.766–0.806)	0.583 (0.549–0.617)	0.724	0.697	0.735	0.512	0.859
	Temporal validation	0.764 (0.742–0.786)	0.561 (0.526–0.596)	0.706	0.673	0.719	0.493	0.846
XGBoost	Testing	0.803 (0.784–0.822)	0.612 (0.578–0.646)	0.742	0.718	0.752	0.536	0.872
	Temporal validation	0.779 (0.758–0.800)	0.584 (0.549–0.619)	0.721	0.692	0.733	0.514	0.857
SVM	Testing	0.761 (0.740–0.782)	0.549 (0.514–0.584)	0.698	0.671	0.709	0.482	0.843
	Temporal validation	0.742 (0.719–0.765)	0.528 (0.492–0.564)	0.681	0.652	0.693	0.467	0.833
k-Nearest neighbors	Testing	0.714 (0.691–0.737)	0.496 (0.461–0.531)	0.658	0.627	0.670	0.438	0.815
	Temporal validation	0.693 (0.669–0.717)	0.473 (0.438–0.508)	0.641	0.608	0.654	0.421	0.803

AUPRC = area under the precision-recall curve, AUROC = area under the receiver operating characteristic curve, CI = confidence interval, NPV = negative predictive value, PPV = positive predictive value.

95% CIs were derived from 1000 bootstrap iterations. Optimal classification thresholds were determined by Youden J statistic.

**Figure 2.**
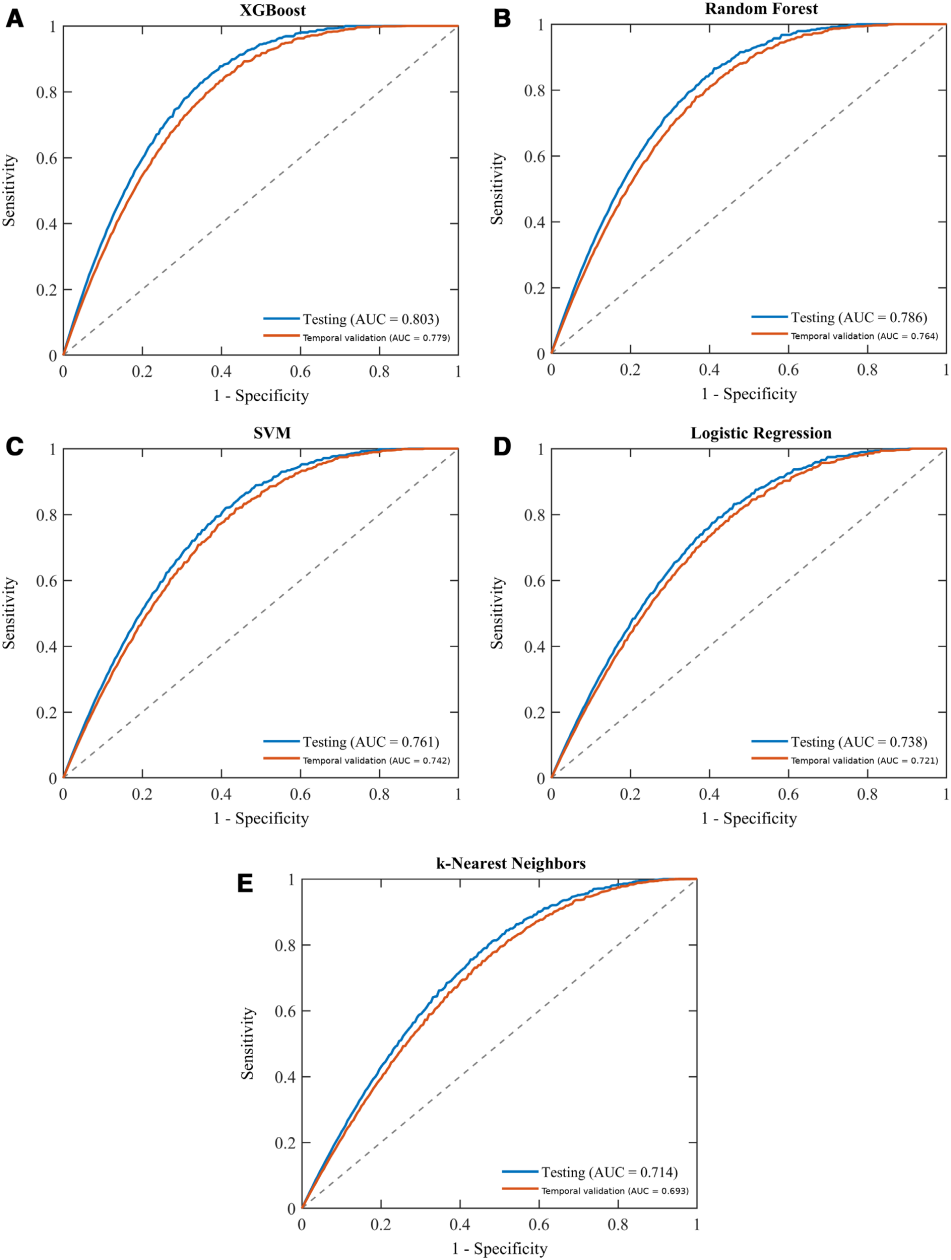
ROC curves for machine learning models. (A) XGBoost. (B) Random forest. (C) SVM. (D) Logistic regression. (E) k-Nearest neighbor. ROC = receiver operating characteristic curve, SVM = support vector machine, XGBoost = extreme gradient boosting.

Figure 2A to E presents ROC curves comparing testing and temporal validation performance for each model. XGBoost demonstrated the highest discrimination, with curves positioned closest to the upper-left corner, while all models exhibited expected performance attenuation in temporal validation, with testing curves consistently above temporal validation curves.

Calibration assessment using the Hosmer-Lemeshow goodness of fit test indicated acceptable calibration for XGBoost (χ^2^ = 11.23, *P* = .189) and logistic regression (χ^2^ = 9.87, *P* = .274), marginally acceptable calibration for random forest (χ^2^ = 14.56, *P* = .068), moderate calibration concerns for SVM (χ^2^ = 18.42, *P* = .018), and poor calibration for k-nearest neighbors (χ^2^ = 28.64, *P* < .001).

**Figure 3.**
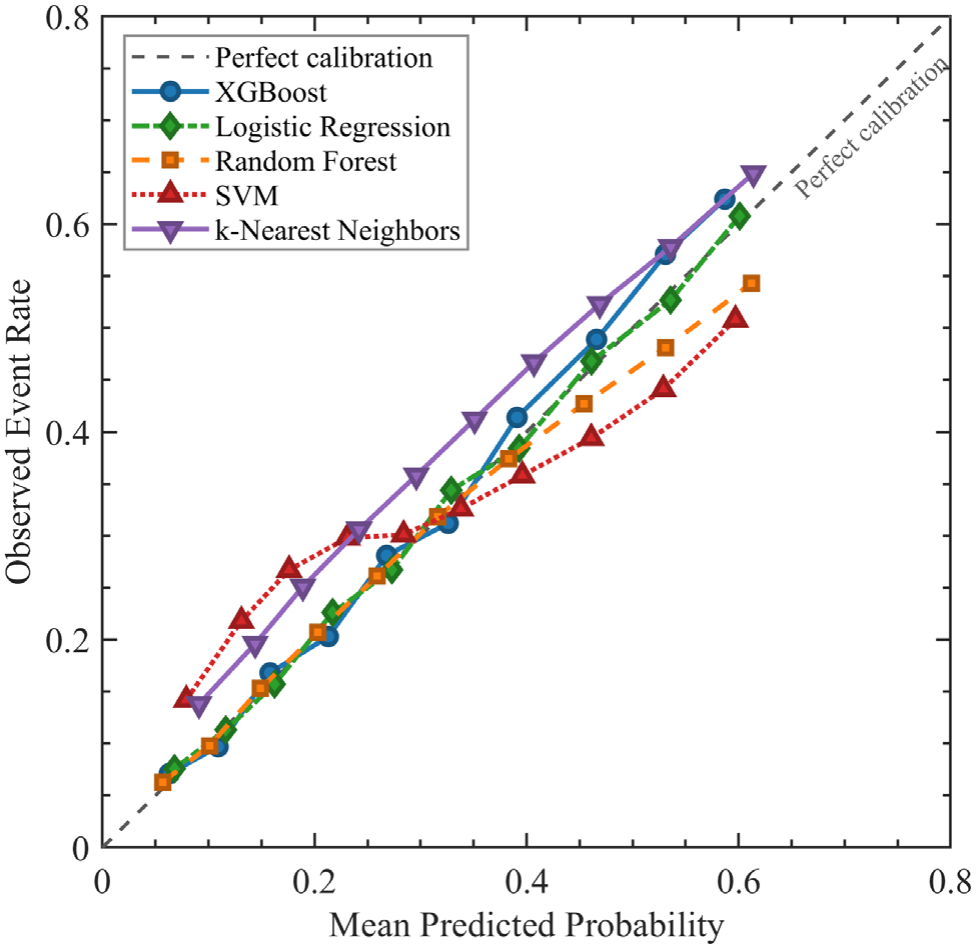
Calibration curves for all 5 machine learning models on the internal testing set. SVM = support vector machine, XGBoost = extreme gradient boosting.

Calibration curves were generated for all 5 models on the internal testing set, as presented in Figure 3.

Figure 3 demonstrates that XGBoost and logistic regression exhibited the closest alignment between predicted probabilities and observed event rates across risk deciles on the internal testing set, with calibration curves tracking near the diagonal reference line throughout most of the probability range. Random forest showed minor overestimation of risk in the upper deciles, while SVM and k-nearest neighbors displayed more pronounced deviation, consistent with their respective Hosmer-Lemeshow findings.

**Figure 4.**
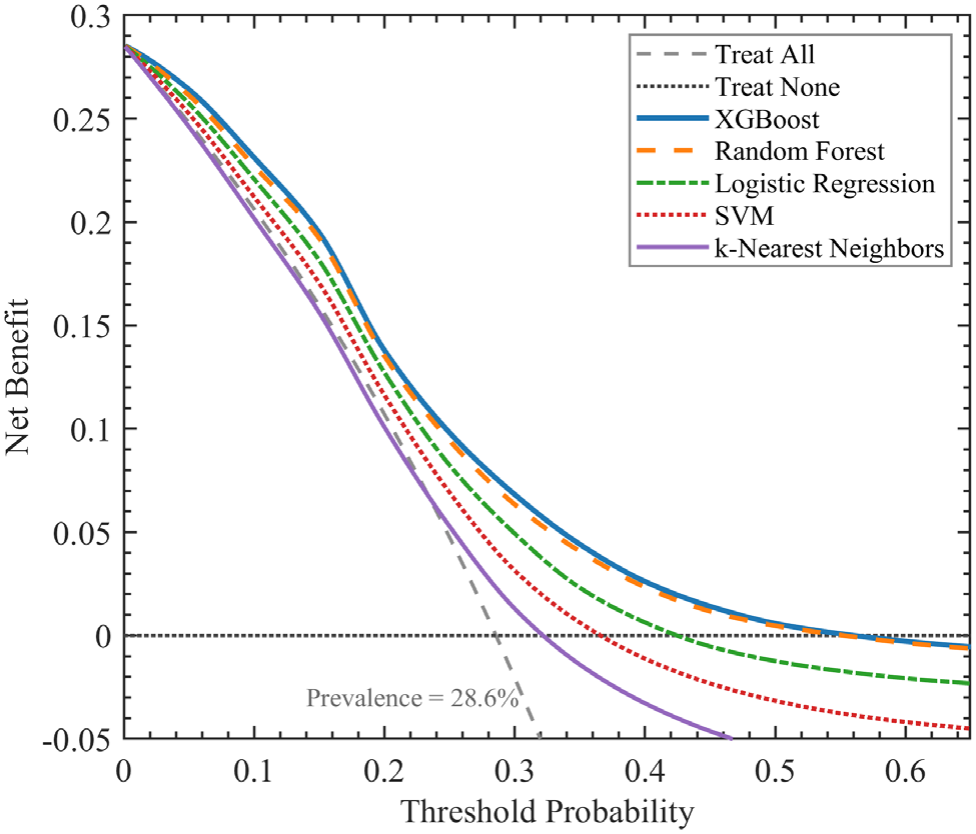
Decision curve analysis for all 5 machine learning models on the internal testing set. SVM = support vector machine, XGBoost = extreme gradient boosting.

To evaluate whether the discriminative and calibration performance of the models translates into actionable clinical benefit, decision curve analysis was conducted across a threshold probability range of 1% to 99%, with results for the internal testing set presented in Figure 4.

Figure 4 shows that across the clinically plausible threshold range of approximately 10% to 55%, XGBoost consistently provided positive net benefit above both the treat-all and treat-none reference strategies, indicating that application of the model to guide preventive surveillance would yield a net clinical advantage relative to nonselective or no intervention. At a threshold probability of 20%, which may represent a reasonable clinical decision point given the population-level incidence of 28.8%, the net benefit of the XGBoost model was 0.138, compared with 0.107 for the treat-all strategy, implying that the use of the model would avoid approximately 3 to 4 unnecessary preventive interventions per 100 patients without sacrificing true case identification relative to treating all patients indiscriminately. Random forest yielded comparable net benefit to XGBoost across most of this range, with the 2 curves remaining closely overlapping between thresholds of 15% and 45%. Logistic regression demonstrated progressively declining net benefit at thresholds above 35%, while SVM and k-nearest neighbors demonstrated notably lower net benefit across the clinically relevant threshold range, with their net benefit curves crossing the treat-none line at approximately 38% and 33%, respectively, indicating diminishing clinical utility at higher decision thresholds.

### 3.3. Key predictors of multimorbidity

**Figure 5.**
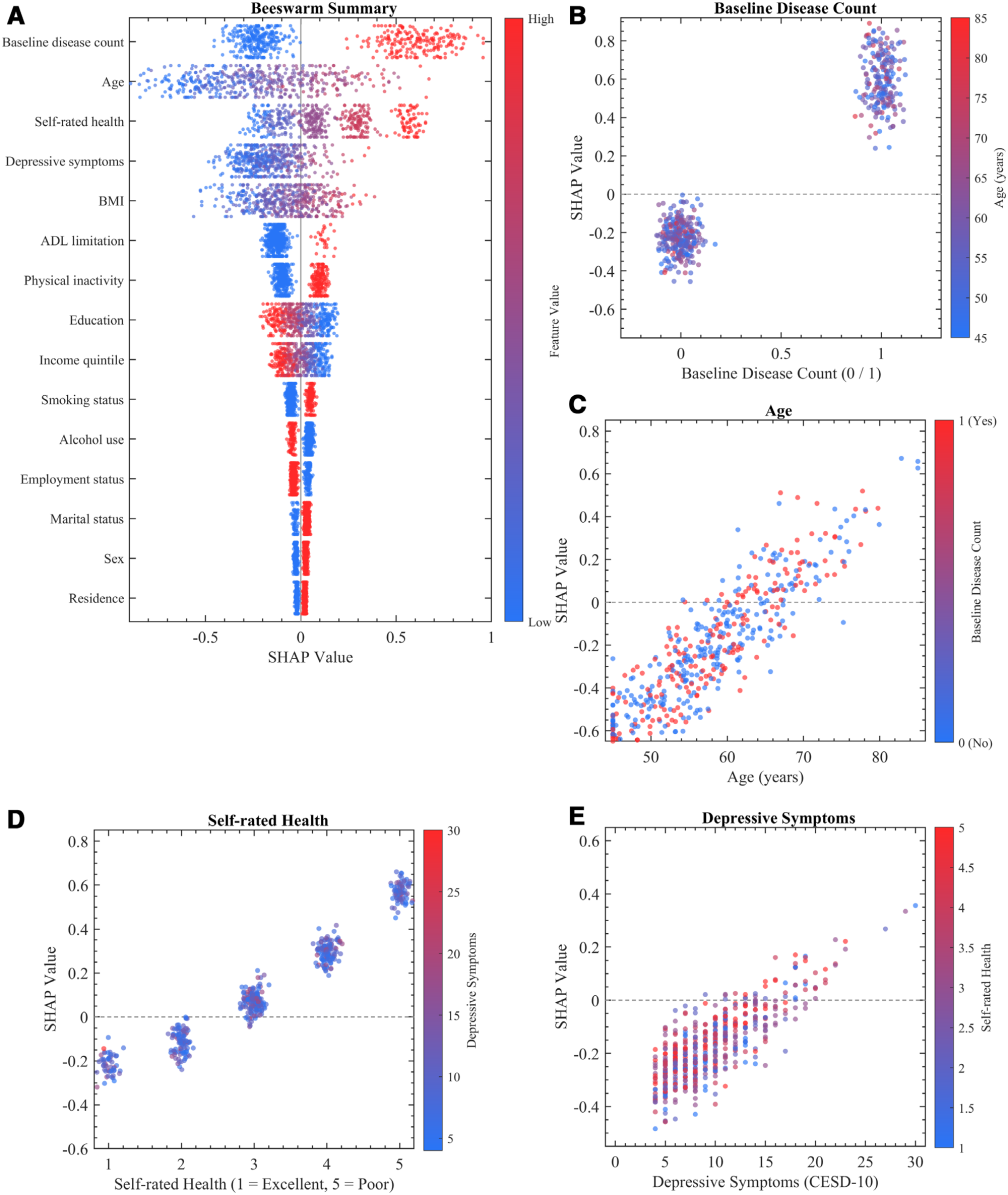
SHAP analysis of the optimal XGBoost model applied to the internal testing set. (A) Beeswarm summary. (B) Baseline disease count. (C) Age. (D) Self-rated health. (E) Depressive symptoms. ADL = activities of daily living, BMI = body mass index, SHAP = SHapley Additive exPlanations, XGBoost = extreme gradient boosting.

Feature importance values were determined using the optimal XGBoost model, with baseline chronic disease status being identified as the most influential predictor (gain-based importance = 0.187), followed by age (0.142), self-assessed health status (0.098), and depressive symptoms (0.084). To provide individual-level interpretability and validate the gain-based rankings through a theoretically grounded framework, SHAP values were computed for all observations in the internal testing set, with results presented in Figure 5.

Figure 5A presents the beeswarm summary plot, in which each point represents a single observation colored by the corresponding feature value, with red indicating higher feature values and blue indicating lower values. SHAP analysis confirmed the predominance of baseline chronic disease status as the single most influential predictor (mean |SHAP| = 0.384), consistent with its gain-based ranking, followed by age (mean |SHAP| = 0.312), self-rated health (mean |SHAP| = 0.226), and depressive symptoms (mean |SHAP| = 0.187). The directional patterns revealed by the beeswarm plot indicate that higher baseline disease burden, older age, poorer self-rated health, and greater depressive symptom severity were each associated with higher SHAP values and thus increased predicted probability of incident multimorbidity, while higher physical activity level and higher household income were associated with negative SHAP contributions reflecting reduced predicted risk. The rank-order consistency between SHAP-derived and gain-based importance rankings was high (Spearman ρ = 0.91, *P* < .001), supporting the robustness of predictor identification across methodological approaches. Figure 5B to E presents dependence plots for the 4 highest-ranked predictors, revealing nonlinear associations between predictor values and their SHAP contributions. A notably steep risk gradient was observed at lower self-rated health scores, where marginal declines in perceived health status corresponded to disproportionately large increases in SHAP contribution. Baseline disease count exhibited a near-threshold effect, with SHAP contributions increasing sharply as the count approached the study definition of multimorbidity, a pattern that is consistent with but also partially reflective of the structural proximity between this predictor and the outcome definition discussed in the limitations.

Health status variables, namely BMI (0.076) and activities of daily living limitations (0.068), occupied the next 2 ranks, followed by moderately predictive lifestyle variables: physical inactivity (0.057), smoking status (0.026), and alcohol consumption (0.021). Socioeconomic factors, like level of education (0.049) and family income (0.043), demonstrated gradients consistent with previous reports on educational and income gradients. The high degree of correspondence between feature importance rankings obtained using XGBoost and random forest modeling methods (Spearman ρ = 0.94, *P* < .001) implies reliable and algorithm-independent identification of influential predictors. Aggregation at the domain level showed that health status variables made the greatest contribution (33.6%), followed by sociodemographic factors (23.4%), status at disease onset (19.3%), socioeconomic factors (13.0%), and lifestyle variables at 10.7%. Subgroup analysis showed stable performance across different strata with regard to age and sex. Specifically, AUROCs were similar among individuals aged 45 to 59 and 60+ years old (0.778 vs 0.812) and among males and females stratified by sex (0.796 vs 0.809). As a sensitivity analysis, the XGBoost model reestimated on complete cases only (n = 6847 after listwise deletion, representing a 19.9% reduction from the primary analytic sample) yielded an AUROC of 0.797 (95% CI = 0.775–0.819) on the internal testing set, consistent with the primary imputed analysis (AUROC = 0.803), supporting the robustness of the MissForest imputation strategy.

## 4. Discussion

### 4.1. Main findings and comparison with previous studies

The present study shows that ML models, specifically XGBoost, have strong discriminatory predictive abilities for incident multimorbidity among mid-life and older Chinese people. The best model had an internal AUROC of 0.803 (95% CI = 0.784–0.822) and a temporal validation AUROC of 0.779 (95% CI = 0.758–0.800). The small relative cross-validation difference of 3.0% reflects acceptable model applicability across recruitment phases. Recent research efforts addressing Chinese elderly populations using ML models for predicting multimorbidity pattern forecasts have indicated that disease pattern extraction and prediction using ML models are feasible and applicable.^[31]^ Together with previous efforts using CHARLS data sets and ML models for forecasting cardiovascular disease, there have been demonstrated capabilities of ML models for disease risk prediction and preventive measures.^[32]^ The 7-year cumulative proportion of 28.8% fits within epidemiological standards, and it was seen that baseline chronic disease status had strong predictive value with importance = 0.187, reflecting evidence that having a single disease status significantly impacts subsequent multimorbidity risk establishment and recognized predictive associations with mortality rates.^[33]^

### 4.2. Implications for prevention and clinical practice

Several predictors identified in this study are potentially modifiable in nature, carrying implications for risk-targeted practice and health policy. The prominent predictive contributions of poor self-rated health (importance = 0.098), depressive symptoms (0.084), elevated BMI (0.076), and physical inactivity (0.057) suggest that individuals exhibiting these characteristics may represent a high-priority subgroup for prospective monitoring and preventive intervention, though the predictive nature of this model does not permit causal attribution, and the clinical impact of targeting these factors would require confirmation through prospective intervention studies. The costs associated with multimorbidity are widely recognized, and people who have multiple chronic conditions have significantly greater healthcare expenses,^[34]^ thus justifying preventive intervention efforts. The cost impact aside, it is recognized that multimorbidity influences health-related quality-of-life changes significantly differently based on the number and type of conditions affecting an individual, and there is systematic proof that there are meaningful changes because of multiple conditions.^[35]^

### 4.3. Strengths and limitations

Strengths include employing a large national cohort with a high 7-year follow-up rate (n = 8552), making comparisons across 5 prediction algorithms, and conducting temporal validation on an independent sample (n = 3218). The high degree of correspondence between rankings of variable importance for XGBoost and random forest models (ρ = 0.94) adds credibility that these variables relate genuinely. Weaknesses include reliance on self-reported physician-diagnosed conditions, which are susceptible to recall and social desirability bias. To partially address this limitation, the study restricted outcome ascertainment to conditions ascertained via physician-defined diagnoses as reported by participants and documented consistently across all CHARLS waves, which provides a degree of standardization relative to purely self-reported symptom data. Nevertheless, residual misclassification cannot be excluded, and the possibility of residual confounding from unmeasured variables remains. Although missing data were handled using MissForest imputation under the missing at random assumption, a complete-case sensitivity analysis confirmed that the primary conclusions were not materially affected by the imputation approach (complete-case AUROC = 0.797 vs primary AUROC = 0.803). Furthermore, certain health-related predictors such as depressive symptoms and self-rated health status may conceptually function as mediators rather than independent predictors in the etiology of multimorbidity. As the present study adopts a predictive rather than causal framework, this distinction was not addressed in the modeling strategy, and the potential mediating roles of these variables warrant examination in future studies employing causal inference methods. The inclusion of baseline chronic disease burden as a predictor for incident multimorbidity warrants careful interpretation, as participants with a higher baseline disease count are structurally closer to the multimorbidity threshold, which may inflate the apparent predictive contribution of this variable. Future studies are encouraged to evaluate model performance with baseline disease status excluded or used as a stratification variable to assess the robustness of the remaining predictors. The lack of interpretability could potentially hinder implementation, but novel techniques exist for interpreting algorithms that promise ways forward.^[36]^

## 5. Conclusions

This study demonstrates that ML models, including XGBoost as an illustrative example, are capable of stratifying risk for multimorbidity among mid-life and older Chinese populations. Internal validation achieved an AUROC of 0.803, and temporal validation an AUROC of 0.779, with good calibration (Hosmer-Lemeshow χ^2^ = 11.23, *P* = .189). Baseline presence and number of chronic conditions, age, self-rated health, and depressive symptoms were seen to be the most important predictors, and health status variables as a set explained 33.6% of variable importance. The prominent predictive roles of poor self-rated health, depressive symptoms, elevated BMI, and physical inactivity highlight potentially actionable characteristics that may inform the design of risk-targeted preventive programs, though causal inference remains beyond the scope of this predictive modeling study. Potential avenues for future research include exploring ways to incorporate biomarkers and developing an interpretable model that would be more feasible within a family practice setting.

## Author contributions

**Conceptualization:** Yu Wang.

**Data curation:** Yu Wang.

**Formal analysis:** Yu Wang.

**Investigation:** Yu Wang.

**Methodology:** Yu Wang.

**Project administration:** Yu Wang.

**Resources:** Yu Wang.

**Software:** Yu Wang.

**Supervision:** Yu Wang.

**Validation:** Yu Wang.

**Visualization:** Yu Wang.

**Writing – original draft:** Yu Wang.

**Writing – review & editing:** Yu Wang.


