# Supplementary material for: Machine learning-based early prediction of multiple chronic disease risk in aging Chinese population: A longitudinal analysis using CHARLS data
Source: Medicine (Baltimore). 2026 Jun 5;105(23):e49175. doi: 10.1097/MD.0000000000049175 (PMC13246033; doi:10.1097/MD.0000000000049175)
Supplement: Supplementary file 1 [file medi-105-e49175-s001.docx]

**Table S1.** Missing data proportions for all 28 candidate predictor variables (n = 8,552)

| **Domain** | **Variable** | **Missing, n** | **Missing, %** |
| --- | --- | --- | --- |
| **Sociodemographic** | Age, years | 0 | 0.0 |
|  | Sex | 0 | 0.0 |
|  | Urban/rural residence | 0 | 0.0 |
|  | Geographic region | 0 | 0.0 |
|  | Marital status | 26 | 0.3 |
|  | Education level | 43 | 0.5 |
|  | Living arrangement | 103 | 1.2 |
| **Socioeconomic** | Health insurance coverage | 51 | 0.6 |
|  | Employment status | 120 | 1.4 |
|  | Health insurance type | 154 | 1.8 |
|  | Pension receipt | 179 | 2.1 |
|  | Per capita household expenditure | 607 | 7.1 |
|  | Household income quintile | 710 | 8.3 |
| **Lifestyle** | Current smoking status | 68 | 0.8 |
|  | Alcohol consumption | 239 | 2.8 |
|  | Sedentary time, hours/day | 325 | 3.8 |
|  | Physical activity level | 393 | 4.6 |
|  | Dietary diversity score | 462 | 5.4 |
| **Health status** | Self-rated health status | 34 | 0.4 |
|  | ADL limitation | 94 | 1.1 |
|  | IADL limitation | 137 | 1.6 |
|  | Depressive symptoms (CESD-10) | 299 | 3.5 |
|  | Cognitive function (MMSE score) | 410 | 4.8 |
|  | BMI, kg/m² | 530 | 6.2 |
|  | Waist circumference, cm | 572 | 6.7 |
|  | Grip strength, kg | 650 | 7.6 |
| **Baseline disease** | Presence of any chronic condition | 0 | 0.0 |
|  | Number of chronic conditions | 0 | 0.0 |

*Missing data were imputed using the MissForest algorithm. Imputation accuracy was assessed via out-of-bag error, yielding rates of 0.127 for continuous variables and 0.089 for categorical variables. ADL, activities of daily living; BMI, body mass index; CESD-10, Center for Epidemiologic Studies Depression Scale (10-item); IADL, instrumental activities of daily living; MMSE, Mini-Mental State Examination.*
